# PE augmented mindfulness: A neurocognitive framework for research and future healthcare

**DOI:** 10.3389/fnhum.2022.899988

**Published:** 2022-08-23

**Authors:** David Sars

**Affiliations:** ^1^Mettaminds.org, Mindfulness Based Projects, Amsterdam, Netherlands; ^2^Centre for Integral Rehabilitation (CIR), Amsterdam, Netherlands

**Keywords:** mindfulness, yoga, aerobic physical exercise, vagal tone, stress buffering, sympathetic withdrawal, cognitive behavioural therapy, protocol development

## Abstract

Various well-controlled studies have suggested that practitioners in mindfulness can be prone to patient drop-out (e.g., due to chronic stress, pathology, cognitive reactivity), despite researchers having identified the underlying mechanisms that link mindfulness to mental health. In this article, a framework for physical exercise (PE) augmented mindfulness is proposed, which posits that consistently practiced PE before meditation can support (early-stage) mindfulness. Neurocognitive research shows PE (aerobic exercises or yoga) and mindfulness to impact similar pathways of stress regulation that involve cognitive control and stress regulation, thereby supporting the proposed synergistic potential of PE augmented mindfulness. Research focused on the psychophysiological impact of PE, showed its practice to promote short-term neurocognitive changes that can promote both cognitive control and the attainment of mindful awareness (MA). In order to chart dose responses required for protocol development, further research will be presented. Together these findings are discussed in light of future research on this multidisciplinary topic, protocol development, mindful walking, and further application in healthcare and beyond.

## Introduction: Mindfulness augmented with physical exercise

Individual and group-based therapeutic interventions increasingly apply mindfulness- based principles and techniques for attention practice. The integration of mindfulness within these therapies is motivated by extensive evidence for its capacity to improve self-regulation (Germer et al., 2016; Guendelman et al., 2017). The development of mindful awareness (MA) allows patients to grow in their ability to regulate attention, emotion, physiology and the environment in response to emotional arousing events or circumstances. Such benefits may, however, be difficult to attain for patients who experience chronic stress; e.g., due to pathology or chronic pain. Even though mindfulness has potential to improve cognitive control (Li et al., 2018; Incagli et al., 2019), its practices and meditations can suffer from the adverse impact of chronic stress on attention regulation (Arnsten, 2009). Indeed, studies on protocolled mindfulness indicate a substantial risk for patient drop-out (Khoury et al., 2013; Li and Bressington, 2019), particularly at an early intervention phase, this to be associated with lower levels of cognitive control (Crane and Williams, 2010; Dobkin et al., 2011), and long-term practice to be required for its cognitive and stress reducing benefits to emerge (Tang et al., 2015; Engert et al., 2017). In mindfulness, the inability to address patient cognitive vulnerability could represent a therapeutic gap in its clinical application.

The complementary application of physical exercise (PE) could help to bridge this gap, when practiced consistently before mindfulness meditation. Mindfulness initially engages the top-down pathway of stress regulation, predominantly via attention practice, and causes slow changes in the neurophysiology of the stress response system. However, to directly reduce symptoms of chronic stress, protocolled mindfulness could be augmented with PE to precede its meditations. Besides PE causing direct improvement in cognitive control (Basso et al., 2015; Pontifex et al., 2021) and reduction of the main stress hormone cortisol (Rudolph and McAuley, 1998; Hill et al., 2008), it may also contribute to patient self-motivation during subsequent meditation. For instance, its practice was found to directly improve mood (Basso and Suzuki, 2017) and reduce cognitive reactivity (Herring et al., 2017; Brand et al., 2018), outcomes that have been linked to improved psychological engagement in mindfulness training (e.g., Banerjee et al., 2018). Therefore, PE may not only promote MA development, it may also do so in early-stage mindfulness.

In clinical health care, the neurocognitive impact of combined practice may not be tuned for optimal MA development. For instance, the Mindfulness Based Cognitive Therapy Protocol (MBCT) (Segal et al., 2002), being a blueprint for many Mindfulness Based Interventions (MBI’s), does practice yoga – but not consistently before its meditation practices. Likewise, the Mental and Physical training (MAP) practices meditation before aerobic training, which was found to cause neurocognitive changes in clinical depressed patients (Alderman et al., 2016). For the treatment of work stress researchers already proposed sequencing exercise and yoga before mindfulness (Sars, 2014; De Bruin et al., 2017), while finding positive results for such an approach (De Bruin et al., 2020). Nonetheless, here it is proposed that existing protocolled mindfulness programs can be adapted, while emphasizing minimal PE dose–responses in relation to underlying working mechanisms. This article, therefore, presents a neurocognitive framework for PE augmented mindfulness, while outlining common and complementary mechanisms of change for mindfulness and PE.

From a neurocognitive perspective the impact of mindfulness on practitioner brain functioning can be seen as a result of experiential learning. The practice of focused attention (e.g., on breath or body awareness) and open monitoring meditation, combined with the daily cultivation of MA, provides a specific context for brain exercise. MA can be described as a non-reactive, i.e., to respond less automatically to thoughts and feelings, and a compassionate, present centered state, which encompasses the ability to pay attention to cognitive processes. Research found mindfulness to stimulate brain regions that are part of the central executive network (CEN) (Bauer et al., 2019), which regulates cognitive control over experience and is highly involved in self-regulation (Seeley et al., 2007). Mindfulness was found to augment connections between a specific brain region in that network, the prefrontal cortex (PFC), and the (cortically lower) emotion processing centers of the midbrain (e.g., the hypothalamus, amygdala). Repeated activation of this top-down pathway can cause a reduction of peripheral physiological stress response cascades, eventually contributing to reduced stress reactivity (Creswell and Lindsay, 2014). One potential way in addressing the impact of PE on mindfulness could be to examine whether or not PE can contribute to similar effects by engaging similar neural pathways. In doing so, PE could serve to prime the neurocognitive processes also implied in mindfulness.

Mindfulness has been also found to alter stress reactivity of cortically lower regions by means of bottom-up regulation. For example, researchers found altered neural connectivity in subcortical brain areas (related to body awareness) amongst long-term meditators to be independent of PFC activation (Van den Hurk et al., 2010; Westbrook et al., 2011). Chiesa et al. (2013) accordingly stated that short term practitioners in mindfulness mainly reduce unpleasant (i.e., stressful) emotions by means of reappraising such an emotion more adaptively, whereas long-term practitioners rather employ the ability to observe it in a more detached manner. This latter step may require a stabilized degree of peripheral psychophysiological health; one that reduces sympathetic activation, but promotes sufficient parasympathetic activation in order to break down (fight, flight, –or-freeze) stress responses (Thayer and Lane, 2000; Creswell and Lindsay, 2014) and sensing ability of inner body signals (i.e., interoception) as a prerequisite for effective emotion regulation (Füstös et al., 2012). In line, eventual reduction of stress hormones through mindfulness shifts the autonomic nervous system (ANS); the ‘restorative’ parasympathetic subsystem (PSNS) becomes more active and less antagonistically excluded by the ‘stress and action based’ sympathetic subsystem (SNS).

The mindful practice of PE has been proposed as a therapeutic alternative for patients who suffer cognitive reactivity to a degree that hinders regular meditation (Russell and Arcuri, 2015). There are good indications that PE primes neurocognition toward improved cognitive control and mind-body awareness. Firstly, PE itself can be described as an attention practice; one that emphasizes attention on breath regulation, psycho-motoric coordination and active monitoring of physiological, psychological and environmental functions. In sports literature this type of focus is often referred to as metacognitive monitoring (Nietfeld, 2003; MacIntyre et al., 2014), which has conceptual overlap with MA. Secondly, and this is clearly related, studies on PE also found practice to promote flow-state experience: a (pleasant) state of cognitive absorption or one of undivided attention to a limited stimulus field, characterized with a fading out of the mind of self-referential thoughts (Kawabata and Mallett, 2011; Peifer et al., 2015). Thirdly, PE practices can trigger a relaxation response (Benson and Proctor, 2011; Luberto et al., 2020) through PSNS activation, which, in turn, has been associated with increased attentional control (Giuliano et al., 2018).

Notably, the direct impact of PE on PSNS activation is closely related to parasympathetic activation of the vagus nerve, which comprises approximately 75% of the PSNS (Robertson et al., 2011). In research, PSNS or vagal tone is measured on a physiological level through heart rate variability (HRV), which measures direct vagus nerve activation potential on a cardiac level (Thayer and Lane, 2000). By directly impacting stress and cardiac physiology, PE can immediately contribute to ANS rebalancing as it increases the potential for PSNS activation. Therefore, as this can contribute to bottom-up regulation, PE is a likely agent to directly promote the practice of mindfulness on a psychophysiological level.

This outline emphasizes PE that promotes flow state experience, by offering clear goals, unambiguous feedback and challenge-skill balance (Csikszentmihalyi, 1998). In line, but also due to the intensity anaerobic practice intermittently places on the nervous system, the current framework focuses on practice with a non-anaerobic signature that is practiced under or near 65% of a practitioner’s capacity. Aerobic exercises, therefore, as practiced in running, cycling, swimming, light bootcamp or dance, as well as yoga poses (yogasanas) are implied in this approach and generally referred to as PE.

Importantly, the mindful practice of yogasanas in most research is often not studied exclusively. While representing the largest proportion in most studied session protocols, yogasanas generally are modulary supplemented by pranayama (yogic breath work), the both of which are aerobically beneficial (Larson-Meyer, 2016; Hota et al., 2017), but also dhyana (meditation) practice. Nonetheless, like in aerobic exercise, the yogasanas themselves can be practiced mindful and with emphasis on breath regulation. For these reasons, in the following, there will be mention of yoga practice unless a study has exclusively focused on yogasanas.

Mindfulness and PE, presenting similarities as well as differences in practice impact, could offer a new approach to mindful stress regulation when combined. Mindfulness practice initially activates the top-down pathway of stress regulation through its mental orientation, only to activate bottom-up stress regulation at a later stage (Chiesa et al., 2013). PE, on the other hand, can be considered an attention practice on mind-body awareness, promotes direct physiological changes that improve stress-regulation, yet often is presented without a protocol that offers psychological guidance. Although PE is less focused on utilizing MA, its practice is expected to promote bottom-up stress regulation and support underlying processes in MA development. It is therefore that PE consistently practiced before meditation is proposed to promote MA in (early-stage) mindfulness, as it improves both practitioner (1) neurocognitive faculties of self- and stress regulation and (2) overall ANS functioning.

In the following, first section, an outline of the PE augmented mindfulness framework will be given. The second section takes a neurocognitive approach, as it follows a stress buffering explanation on mindfulness. PE is proposed to impact similar (top-down) mechanisms by which mindfulness impacts stress regulation, which will be further examined based on mindfulness, aerobic exercise and yoga research. The third section takes a psychophysiological approach, by examining key mechanisms through which aerobic exercise and yoga can directly contribute to (bottom-up) neurocognition. The fourth section further discusses dose–response characteristics for PE to be effective and how these can be best implemented into protocolled mindfulness. Implications of the current framework will be discussed in the fifth section.

## PE augmented mindfulness as a framework

### Neurocognitive impact

From a neurocognitive perspective, attention practices can contribute to practitioner ability to adopt and retain MA. In order to explore this contribution, both mindfulness and PE should be examined on their neurocognitive impact. Therefore, a brief review of the brain networks involved in a cycle of attention will be given. These networks are best described as constructs, defined by interacting brain regions, that correlate with each other and that are distinct from other networks. Hasenkamp and Barsalou (2012) aimed to chart the networks involved by asking participants to perform a breathing meditation while under a fMRI scanner. When participants realized their mind had wandered, they were required to press a button and return their focus back to their breath. These researchers managed to chart four intervals in a cognitive cycle: In the first phase, of (resumption of) focused attention, the dorsolateral prefrontal cortex of the CEN showed a high level of activity. In the second phase, when there was an episode of mind wandering, there was an increase of activity in the default mode network (DMN). The DMN plays a role in generating internal models of the world, based on long-term memories and includes self-referential processing and mentalizing processes. In the third phase, activity in the salience network (SN) increases during awareness of mind wandering. In the fourth phase, attention is reoriented or detached from any distracting stimulus – which again shows increased CEN activity.

Researchers describe MA as an advanced degree of neurocognitive functioning and have specifically linked this awareness to the CEN (Shimamura, 2000; Jankowski and Holas, 2014). This network activates during tasks that require cognitive control over experience; e.g., during emotion regulation by modulating related brain structures (Ochsner and Gross, 2005), monitoring conflicts and executive attention (Van Veen and Carter, 2002), task switching activity between different brain networks (Sridharan et al., 2008) and the selection of appropriate signals and suppressing inappropriate signals (Fernandez-Duque et al., 2000; Stuss et al., 2001; Pannu and Kaszniak, 2005). In their mindfulness stress buffering account, Creswell and Lindsay (2014) describe the CEN to play a central role in the neural pathways that are activated through mindfulness. Though in part based on preexisting research, the model specifies mindfulness to produce measurable neurocognitive effects.

The mindfulness stress buffering account outlines two stress-processing pathways in the brain that are altered. Firstly, mindfulness increases the recruitment of prefrontal regulatory regions that may inhibit activity in stress-processing regions (*a “top-down”* regulatory pathway), by increasing activity and functional connectivity of CEN network components (e.g., the dorsolateral prefrontal cortex – PFC) that modulate other stress-processing regions – to include DMN and SN components. Through this pathway noradrenergic release (both norepinephrine and epinephrine) is regulated both in the brain and SNS via the sympathetic-adrenal-medullary (SAM) axis. Secondly, mindfulness may also have a direct modulating effect on stress-processing regions (*a “bottom-up”* reduced-stress-reactivity pathway), by decreasing activity yet improve functional connectivity in neural (e.g., DMN) regions that gate stress responses through to the hypothalamic–pituitary–adrenal (HPA) axis (e.g., the amygdala) – which regulates cortisol release. The stress buffering account points out that these changes cause reduced peripheral physiological stress-response cascades in the SAM- and HPA- axes. It is further described that a shift toward this functionality promotes parasympathetic activity, non-reactivity and physiological changes that contribute to ANS health.

The activation of top-down and bottom-up processing as prompted through mindfulness, requires time to cause corresponding neurocognitive changes. Research indicates that top-down regulation through the inhibitory function of the PFC causes decreased SAM- but also to a lesser extent HPA axis activation (Ochsner et al., 2009; Arnsten et al., 2015). PSNS activation, however, is strongly affected by reduced cortisol through decreased HPA axis activation – which through mindfulness may require some time to be attained. One mechanism described in the stress buffering account is that SAM axis activation can be altered either through (1) reduction of SNS activation (which impacts the release of norepinephrine and epinephrine) or (2) PSNS activation, which can brake SNS fight or flight stress responses via the vagus nerve (Thayer and Lane, 2000).

**FIGURE 1:**
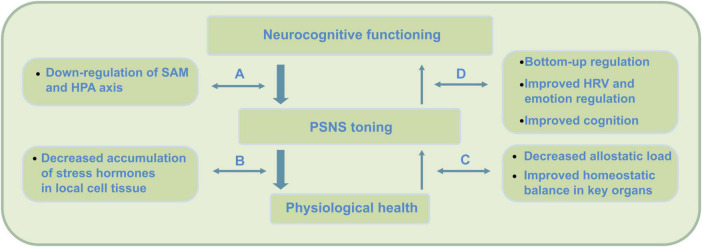
Top-down (stress) regulation pathways engaged in mindfulness practice. Neurocognitive health requires the two subsystems of the autonomic nervous system (ANS) to be in balance; the ‘action or stress based’ sympathetic nervous system (SNS) and the ‘restorative’ parasympathetic nervous system (PSNS). Overactivation of the sympathetic SAM and HPA axes can cause ANS dysregulation, as the PSNS becomes less active due to accumulation of stress hormones such as cortisol. Mindfulness practice initially activates a process of top-down regulation through the inhibitory function of the PFC that causes a decrease (i.e., down-regulation) of SAM but also to a lesser extent HPA axis activation (Ochsner et al., 2009; Arnsten et al., 2015). PSNS or vagal tone, however, is strongly affected by reduced cortisol through decreased HPA axis activation – which through mindfulness may require some time to be attained. Slow reduction of cortisol through mindfulness eventually enables the ANS to extend self-regulation and somatosensory processing into awareness – which, for instance, supports mentalization processing. While mindfulness initially engages top-down regulation during practice, through its meditations, findings suggest a shift to a larger use of bottom–up regulation during later stages of practice (e.g., Chiesa et al., 2013). These top-down effects cause (A) reduced release of sympathetic hormones such as catecholamines and cortisol, (B) decrease of accumulation of these compounds, (C) increase of parasympathetic activity, and (D) increased bottom-up regulation through increased neurovisceral feedback, interoception and cognition.

Importantly, as an attention practice, PE can at least be expected to train similar brain networks as well as target regions involved in the constitution of MA. While the stress buffering account both describes neurocognitive as well as health effects, PE augmented mindfulness proposes that this approach also delineates commonalities in how PE impacts the stress processing pathways described (see Figure 1).

Taken together, PE can be expected to impact similar neural pathways when compared to mindfulness, which may lead to comparable experiential learning in relevant brain regions as well as changes in SAM and HPA axis activation. While such changes cannot be expected after a single-bout of practice, study outcomes on repeated practice could indicate the direction and extent to which PE primes neurocognition in order to directly promote meditation in early-stage mindfulness.

### Psychophysiological impact

The neurocognitive activation due to PE prompts ANS components and corresponding physiological changes. Researchers on direct effects of PE describe PSNS and SNS interplay on all levels of workload, rather than a direct decline of PSNS activity when workload increases (Borresen and Lambert, 2008; White and Raven, 2014). During exercise SAM axis mobilization contributes to the activation of the cardiovascular system, while the HPA axis activates glucocorticoid (notably cortisol) and energy metabolism (Charmandari et al., 2005; Armstrong et al., 2022). Importantly, mobilization of the cardiovascular system involves constriction of blood vessels (pressor reflex) and adjustment of peripheral vascular resistance (baroreflex), which contribute to the resetting of the heart rate control during exercise, but require vagal activity as a modulating agent (Gourine and Ackland, 2019). The mobilization of glucocorticoids regulates carbohydrate, protein and fat metabolism, and is involved – for instance – in the immune responses during inflammation (Dunford and Riddell, 2016). Prolonged activation of these axes through PE results in temporal reduction of stress hormones during practice (e.g., for cortisol, see Caplin et al., 2021), as these become part of the metabolic cycles that become active.

The physiological processes triggered during PE already can be described to promote neurocognitive functioning. Notably, increased cardiovascular activity causes an increased blood flow supply to neural tissue all over the body (particularly those brain regions activated) with nutrients and other biomolecules, promoting overall cell metabolism and neurophysiology (Schmalzl et al., 2015; Heijnen et al., 2016; Gaitán et al., 2019; Liu et al., 2019). These processes also cause the release of biomolecules that promote cognition, such as neurotransmitters related to the formation of new neurons through neurogenesis (e.g., insulin-like growth factor 1) or improvement in learning and memory processes (e.g., body derived neurotrophic factor) (Gligoroska and Manchevska, 2012; Rothman et al., 2012; Jeon and Ha, 2017). Also, with sufficient cardiovascular activation during practice, the corresponding vagal activation can cause a temporal decrease in stress reactivity (Huang et al., 2013; Godoy et al., 2018) and directly contribute to stress regulation on a cardiac level. These studies, taken together, indicate PE to directly impact cell physiology as well as vagal signal processing.

Importantly, following the cessation of PE, these physiological processes promote overall PSNS activation and neurocognition. At this stage, researchers have observed a coordinated interaction of PSNS re-activation and SNS withdrawal, with PSNS re-activation occurring faster, and therefore playing the more important role in the early deceleration of heart rate (Javorka et al., 2002; Kannankeril and Goldberger, 2002; Kannankeril et al., 2004; Pierpont and Voth, 2004). Examination of the parasympathetic effect on heart rate recovery (HRR) found parasympathetic reactivation to occur rapidly in the first minute of recovery (Kannankeril and Goldberger, 2002; Kannankeril et al., 2004), to increase until 4 min and then remain constant until 10 min (Kannankeril et al., 2004). HRR after maximal exercise is slower than after submaximal exercise in healthy individuals, and is attributed to the sympathetic nervous system being stimulated significantly more during maximal exercise. The outcomes indicate (temporal) PSNS activation directly after PE as a mechanism of recovery. Importantly, as this occurs directly after PE, these findings also point out temporal SAM axis stress buffering potential through parasympathetic vagal activation as well as temporal sidelining of HPA axis activation downstream effects.

Other research on post-PE effects indicate practice to alter the process of adaptation that helps the body to maintain ANS homeostasis. This process is termed as allostasis, for which a low measure is indicative of PSNS activation potential. Allostatic load is calculated as a composite measure of neuroendocrine, neurophysiological, anti-inflammatory and metabolic biomarkers (McEwen, 2000; Guidi et al., 2020). While addressing both cognitive and health effects, impact studies found a significant degree of psychophysiological overlap between aerobic exercises and yoga (Ross and Thomas, 2010), with these practices each to contribute to a reduction in allostatic load (D’Alessio et al., 2020). To alter these larger ANS subsystems significantly, long-term practice could be required for these practices. Nonetheless, research thus far indicates that direct physiological changes due to PE, albeit temporal, can already promote PSNS activation substantially.

**FIGURE 2:**
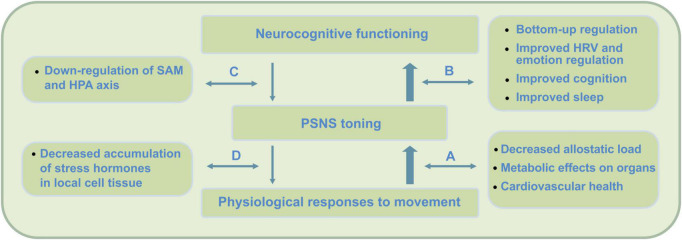
PE physiological and bottom-up short-term impact on neurocognition. Direct effects of PE include improved neurocognitive functioning due to improved parasympathetic nervous system (PSNS) or vagal tone, changes in physiology, as well as postponed neurorestorative effects through improved quality of sleep. In contrast to mindfulness, research indicates these changes to be strongly moderated by psychophysiological factors. By not exceeding the intensity threshold (around 65% VO_2_max) during PE, which causes accumulation of stress hormones (e.g., Hill et al., 2008), this impact is expected to complement mindfulness practice at an early stage. PE has been found to cause direct physiological changes that support key-mechanisms in meditation practice and MA development. Firstly, PE causes temporal cognitive improvements that can contribute to cognitive processes involved in meditation. Secondly, by causing temporal post-exercise PSNS reactivation and SNS withdrawal, stress buffering potential can be improved. By direct increase of PSNS or vagal tone, during subsequent meditation, self- and emotion regulation processes can be promoted. These psychophysiological and bottom-up regulation effects occur though (A) physiological processes that promote parasympathetic health, (B) increased bottom-up regulation effects through increased neurovisceral feedback, interoception and cognition, (C) improved top-down regulation, and (D) reduction of stress hormones on a cellular level – offering potential for increased feedback sensitivity during stress and somatic awareness.

Importantly, research on the direct psychophysiological impact of PE shows it to directly support key-mechanisms that underlie meditation practice and MA development. Firstly, PE causes temporal cognitive improvements as it promotes underlying neurophysiology. Through this, during subsequent meditation, cognitive processes involved in the cycle of attention can be enhanced. Secondly, by causing temporal PSNS reactivation and SNS withdrawal, PE directly contributes to reduced stress reactivity and stress buffering potential. As it improves PSNS or vagal tone, during subsequent meditation, self- and emotion regulation processes (e.g., during mind wandering) can be enhanced. In the current framework, PE is therefore proposed to directly promote meditation practice and early-stage mindfulness (see Figure 2).

Taken together, to provide an overview for this framework, Table 1 lists neurocognitive and physiological benefits of aerobic exercise and yoga.

**TABLE 1:** PE augmented mindfulness: aerobic exercise or yoga practice impact supporting early-stage mindfulness.

	*Aerobic exercise^1^*	Yoga^2^
***Single-bout psychophysiological impact***		
Task positive cognition	Attention, processing speed, executive functioning, memory functioning are improved	Attention, processing speed, executive functioning, memory functioning are improved
HRR – PSNS activation and SNS withdrawal	Post-exercise effects have been measured that indicate stress-buffering potential	The extent to which practice causes cardiovascular activation (also between poses) is expected to produce similar effects – stress buffering potential would be implied
HRV – Vagal tone (and emotion regulation)	Indices of HRV show higher outcomes – which reflects stress buffering potential	Indices of HRV show higher outcomes – which reflects stress buffering potential
Allostatic load reduction	Indications for reduced allostatic load are present – notably direct cortisol reduction	Indications for reduced allostatic load are present – notably direct cortisol reduction
Neurorestorative potential due to improved sleep quality	Indications for improved sleep quality	Indications for improved sleep quality

***Long-term (repeated single bout) impact on neurocognition***		
HPA axis changes	Improved SN (amygdala) functional connectivity	Changes in SN (amygdala) activation
SAM axis (indirectly measured)	Improved CEN functional connectivity and increased gray matter in CEN regions	Improved CEN functional connectivity and increased gray matter in CEN regions
Experiential learning effects	Indications of increased non-reactivity, executive functioning (notably memory) and emotion processing	Indications of increased non-reactivity, executive functioning, emotion processing and somatic awareness

PSNS, parasympathetic nervous system; SNS, sympathetic nervous system; HRR, heart rate recovery; HRV, heart rate variability; HPA axis, hypothalamic–pituitary–adrenal axis; SAM axis, sympathetic–adrenal–medullary axis; SN, salience network; CEN, central executive network.

^1^While aerobic exercise and yoga have significant overlap in term of practice impact, differences in physiological and neurocognitive (i.e., experiential learning) effects can also be expected.

^2^Most studies on yoga practiced both asana’s and meditation. The direct impact of such practice may be notably reflected in physiological changes. In the long-term, however, such practice may not only cause experiential learning unique to yoga, but also broader effects attributable to combined practice.

## Experiential learning leads to improved self-regulation

In this section, mindfulness, aerobic practice and yoga will be further examined on their neurocognitive impact and degree of impact on the mechanisms of stress buffering. For comparison of the experiential learning that results from each practice, there will be a focus on two methods of research. Firstly, fMRI allows researchers to focus on functional connectivity between brain networks as a (indirect) measure for brain network efficiency that results from learning. Such research could help determine how these practices impact the main networks, as described by Hasenkamp and Barsalou (2012) as well as their potential for stress buffering (Creswell and Lindsay, 2014). Secondly, magnetic resonance imaging (MRI) can focus on measurement of structural changes in the brain’s architecture, such as gray brain matter volume. Research outside the field of mindfulness already found targeted brain regions to show increased gray matter depending on the experience of learning itself; e.g., cognitive skills in mirror reading training (Ilg et al., 2008) abstract information processing during study (Draganski et al., 2006) or motor skills during the practice of juggling (Draganski et al., 2004; Driemeyer et al., 2008). For the practice of mindfulness and PE such findings could reflect similar learning processes.

In the following paragraphs research on experiential learning through mindfulness, aerobic exercise and yoga will be discussed. Given the divergence in research literature, each of these practices will be discussed separately. Tables 2, 3, respectively, list obtainable experiential learning effects as neurocognitive outcomes – which were found to qualitatively vary amongst each practice – as well as study characteristics.

**TABLE 2:** Mindfulness meditation: experiential learning effects found and characteristics of included neurocognitive studies.

Study ID code	Neuro-cognitive outcomes	Cognitive impact based on testing	Physiological measures	Design	Participants	Practice type	Sample sizes
	*Functional connectivity*						
Brewer et al., 2011	DMN (PCC) – CEN (dlPFC) improved functional connectivity at (base line) resting state DMN (PCC) – SN (dACC) improved functional connectivity at (base line) resting state DMN (PFC, mPFC) regions are less active at (base line) resting state and during meditation	No	No	Case control matched	Non-clinical	>10 years and an average of 10,565 ± 5,148 h of mindfulness meditation vs. naïve controls	Meditators: *n* = 12 Controls: *n* = 12
Doll et al., 2015	DMN (PCC) – SN (insSN) improved functional connectivity at resting state	FMI correlates negatively with resting state network coupling	No	Correlation study: MA and functional connectivity – no controls	Non-clinical	2 weeks of daily 20-min attention to breath training for meditation naïve participants	Meditators: *n* = 26
Kilpatrick et al., 2011	DMN (dmPFC) – SN (dACC) improved functional connectivity at resting state	MAAS score higher in MBSR group	No	Waitlist control group design	Meditation naïve healthy adult women	8 weeks MBSR training vs. wait list control group (active control)	Mindfulness: *n* = 17 Relaxation: *n* = 15
Taren et al., 2015	DMN (sgACC) – SN (amygdala) improved functional connectivity at resting state	PSS correlates negatively with resting state decoupling	Cortisol correlates negatively with resting state decoupling	Single-blind randomized controlled trial (RCT)	Stressed unemployed community adults	3-day intensive mindfulness meditation training vs. relaxation training without a mindfulness component (active control)	Mindfulness: *n* = 19 Relaxation: *n* = 16
	*Increased gray mass in brain regions related to:*						
Vestergaard-Poulsen et al., 2009	Cardiorespiratory control, self-awareness, executing memory retrieval.	No	No	Matched group design	Non-clinical	Highly experienced meditators vs. naïve controls	Meditators: *n* = 10 Controls: *n* = 10
Hernández et al., 2016	Cardiorespiratory control, self-awareness, executing memory retrieval.	No	No	Matched group design	Non-clinical	Highly experienced meditators vs. naïve controls	Meditators: *n* = 23 Controls: *n* = 23
Hölzel et al., 2011	Learning, memory processing, emotion regulation, self-referential processing and perspective taking	No	No	Waitlist control group design	Non-clinical	8 weeks MBSR training vs. wait list control group	Meditators: *n* = 18 Controls: *n* = 17
Luders et al., 2009	Emotion regulation and response control	No	No	Matched group design	Non-clinical	Experienced meditators vs. naïve controls	Meditators: *n* = 22 Controls: *n* = 22

DMN components: PCC, posterior cingulate cortex/precuneus; mPFC, medial prefrontal cortex; dmPFC, dorsomedial prefrontal cortex; sgACC, subgenual anterior cingulate cortex. SN: dACC, dorsal anterior cingulate cortex; insSN, insula salience network; CEN: dlPFC, dorsolateral prefrontal cortex. FMI: Freiburg Mindfulness Inventory; MAAS: Mindful Attention and Awareness Inventory; PSS: Perceived Stress Scale.

**TABLE 3:** Physical exercise: experiential learning effects found and characteristics of included neurocognitive studies for aerobic practice and yoga.

Study ID code	Neuro-cognitive outcomes	Cognitive/behavioral impact based on testing	Physiological measures	Design	Participants	Practice type	Sample sizes
*Aerobic practice*	*Functional connectivity*						
Voss et al., 2010a	DMN (PCC, MFG, FMC, MTG, PHG) improved intra functional connectivity	Spatial working memory, set-shifting, task-shifting improved	Oxygen uptake, heart rate and blood pressure as a measure of aerobic fitness	Within-subject test design	Non-clinical elderly adults and young adults	The elder group participated in graded maximal testing on a motor driven treadmill *The young adult control group was not tested	Elderly: *N* = 120 Young adults: *N* = 32
Voss et al., 2010b	DMN (MTG, PHG, LOC, MFG) improved intra functional connectivity CEN (RALPFC, PFC) improved intra functional connectivity	Improved executive functions	Oxygen uptake, heart rate and blood pressure as a measure of aerobic fitness	Randomized controlled trial	Non-clinical elderly adults and young adults	The elder group participated in 1 a year intervention trial: aerobic walking group vs. stretching and toning program *The elderly group was randomly assigned to one of two conditions *The young adult control group did not participate in practice	Elderly: *N* = 65 Young adult control group: *N* = 32
Chen et al., 2019	DMN (Parahippocampus, subgenual cingulate) – SN (amygdala) improved functional connectivity during emotional perception task	STAI correlates low with improved functional connectivity through running IPAQ high score mediated this relationship	VO_2_max measurement was used for practice intensity guidance	Within subject crossover design	Non-clinical subjects	Within subjects running and walking sessions were counterbalanced with a 1 week interval between practices. *12-min running session before emotion perception task during a 20-min fMRI scan *12-min walking session before emotion perception task during a 20-min fMRI scan	Young adults: *N* = 39
*Aerobic practice*	*Increased gray mass in brain region(s) related to:*						
Killgore et al., 2013	Spatial memory, learning processes, executive functioning	No	No	Correlational research design	Non-clinical early to middle adult ranging from 18 to 45 years of age	Exercise per week as measured in minutes per week	Adults: *N* = 61
Wittfeld et al., 2020	Memory, emotion, stress-processing, executive functioning	No	Peak oxygen uptake, oxygen uptake at the anaerobic threshold, and maximal power output.	Study on two independent cohorts. Correlational research design	Non-clinical early to elderly adult ranging from 21 to 84 years of age	All participants participated in a cycling exercise based on a modified Jones protocol	Adults: *N* = 2103
Yoga	*Functional connectivity*						
Froeliger et al., 2012a	Different CEN activation patterns – improved functional connectivity	Improved emotional processing	No	Matched group design	Non-clinical early to middle adult ranging from 18 to 55 years of age	>3 years, 3 to 4 times per week, >45 min per day yoga + mediation vs. naïve controls	Yogi’s: *n* = 7 Controls: *n* = 7
Gothe et al., 2018	Improved CEN efficiency	Improved working memory	Cardiorespiratory fitness (used as control over all conditions)	Matched group design	Non-clinical adults between the of 19 and 58 (mean 35.7 years of age)	>3 year, >3 days per week, at least 1 h per day yoga vs. naïve controls	Yogi’s: *n* = 13 Controls: *n* = 13
Yoga	*Increased gray mass in brain region(s) related to:*						
*Gotink et al., 2018*	Fight-flight responses: right Amygdala	*No*	*No*	*Cohort study with repeated measures*	*Large non-clinical population cohort with elevated stress*	>5 years yoga + meditation vs. non-practitioners (no or <5 years of practice)	*Yogi’s: N = 218* Controls: *N* = 2179
Villemure et al., 2015	Visual and sensory processing, somatosensory awareness, self-referential, memory processing, cortical responsiveness	No	No	Matched group design	Non-clinical adults (mean 37 years of age)	Experienced yoga practitioners vs. naïve controls	Yogi’s: *n* = 14 Controls: *n* = 14
Froeliger et al., 2012b	Higher order cognitive control, motor control	No	No	Matched group design	Non-clinical early to middle adult ranging from 18 to 55 years of age	>3 years, 3 to 4 times per week, >45 min per day yoga + mediation vs. naïve controls	Yoga’s: *n* = 7 Controls: *n* = 7

DMN components: PCC, posterior cingulate cortex; MFG, middle frontal gyrus, FMC, frontal medial cortex; MTG, middle temporal gyrus; PHG, parahippocampal gyrus; LOC, lateral occipital cortex; sgACC, subgenual anterior cingulate cortex. CEN: RALPFC, right anterior prefrontal cortex; PFC, prefrontal cortex. STAI, State-trait Anxiety Inventory; IPAQ, International Physical Activity Questionnaire.

### Mindfulness and experiential learning

Research shows that practicing mindfulness causes experiential learning indicating improvement in self-regulation. Its practice was found to lead away from extreme self-referencing and mind-wandering (such as often present in strong rumination and worry) toward a state of non-reactivity. For example, early controlled studies found structural mindfulness practice to improve resting state functional connectivity between DMN (posterior cingulate cortex) and CEN (dorsolateral prefrontal cortex) as well as DMN and SN regions – whereas core nodes of the DMN were found to be less active (Brewer et al., 2011; Doll et al., 2015). Notably, such findings indicate that those who practice mindfulness show a negative association in resting state connectivity between the DMN and SN (Kilpatrick et al., 2011; Doll et al., 2015). Researchers in these studies attributed the results to a greater overall awareness, or sensitivity, to attentional shifting as a result of mindfulness. It was argued that this sensitivity directly relates to non-reactivity, as practitioners typically learn to shift attention back during mind wandering before a stress response is fully triggered. Notably, a 3-day intensive mindfulness retreat was found to already alter stress related amygdala resting state functionality, to lead to a negative correlation between the DMN and SN (amygdala), and a decrease in cortisol amongst practitioners (Taren et al., 2015). These findings show mindfulness to cause changes in network functioning that are consistent with MA development.

Similarly, research also found structural brain changes as a result of mindfulness practice. MRI research found that practitioners showed increased gray matter in brain-stem regions related to cardiorespiratory control relative to non-meditators (Vestergaard-Poulsen et al., 2009). Interestingly, reflecting core practice in mindfulness, other researchers found increased gray matter in CEN regions related to executive control (Hernández et al., 2016) and DMN regions related to self-referential processing, memory processing, emotion regulation, perspective taking and response control (Luders et al., 2009; Hölzel et al., 2011).

The neurocognitive effects found for mindfulness are consistent with the mechanisms described in the stress buffering account (see Table 2). In support of the bottom-up (reduced-stress-reactivity) pathway mindfulness showed corresponding neurocognitive changes – indicating altered HPA activation. Taren et al. (2015) even showed its practice to cause short-term changes in subcortical stress processing regions (e.g., the amygdala) and significant overall decline in cortisol – indicating potential for short-term parasympathetic activation and bottom-up neurocognitive changes. This latter finding does not represent the impact of protocolled mindfulness *per se*, as a 3-day intensive retreat may not durably accommodate the psychological discipline of MA integration. It does indicate mindfulness to cause short-term changes, even though this may depend on factors such as practice intensity and practitioner mental or physical state. In support of the top-down (regulatory) pathway, research also shows mindfulness to improve CEN functional connectivity and practitioner MA – which points to altered SAM axis activation. In addition, the neuroimaging studies also report on practitioner increase in gray mass in brain regions expected to change, when shifting from overt self-referencing toward a more balanced state of MA. The current results were based mainly on long-term or protocolled practice and support the overall finding that mindfulness promotes self-regulation.

### Physical exercise and experiential learning

#### Aerobic exercise

Research shows that aerobic exercise causes experiential learning in domains of cognitive control, memory functioning and emotion regulation. One study focused on the relationship between aerobic fitness, DMN functional connectivity and cognitive performance in the aging brain (Voss et al., 2010a). The data obtained from healthy elderly adults showed that increases found in functional intra DMN connectivity could be described as a function of aerobic fitness level. Functional connectivity within the DMN was also found to mediate the relationship between fitness and cognition. This pertained to tasks such as spatial working memory, set-shifting and task switching – which all rely on executive functioning. These researchers explained that aerobic fitness training may lead to increased DMN connectivity and, in turn, provides a pathway for improved executive function. Another study found that aerobic training increased functional connectivity between aspects of the frontal, posterior and temporal cortices within the DMN and CEN. Here, practice was found to be associated with increased functional connectivity, most notably between the hippocampal area and those in the prefrontal cortex (Voss et al., 2010b). These improvements in network behavior are indicative of improved executive functioning, but yet may also be associated with altered emotion regulation. One study found, relative to walking, that aerobic exercise promoted altered brain connectivity during an emotional perception test (Chen et al., 2019). Although this relationship was mediated by habitual physical activity, the aerobic exercise group showed a negative correlation between regions of the DMN and SN (amygdala) during the processing of fear.

Studies also indicate aerobic exercise to cause structural brain changes. In accordance with its positive impact on cognition, practice was found to increase gray matter in DMN regions (hippocampal) related to memory processing, emotion and stress regulation, CEN regions (orbitofrontal cortex) that relate to executive functioning, and cerebellar regions related to emotion regulation and motor control (Killgore et al., 2013; Erickson et al., 2014; Wittfeld et al., 2020).

The experiential learning impact of aerobic exercise shows a substantial degree of consistency with the stress buffering account. Notably, by focusing on long-term practice, also by incorporating measurement of physical fitness, the moderating impact of physical fitness on both neurocognitive and cognitive outcomes was revealed. These studies measured improvements in network functional connectivity and executive functioning, indicating improvement in self-regulation. Importantly, the finding that aerobic exercise caused improved functional connectivity between DMN and SN components, and that physical fitness level moderated this in regard to fear reduction (Chen et al., 2019), indicates its practice to impact bottom-up processing and alter HPA activation. The findings on improved intra-CEN connectivity as well as increased gray mass in CEN regions also indicate, albeit indirect, improved self-regulation potential through top-down processing and altered SAM activation. Future empirical support – and this may account for yoga research as well – could further be generated from similar methodologies that include cognitive, behavioral and physiological measures, the use of larger sample sizes, by further focusing on long-term versus short-term effects, and by studying the differential impact of different forms of aerobic exercise.

### Yoga

Research shows that practicing yoga causes experiential learning in domains of cognitive control and emotion regulation. One early controlled study by Froeliger et al. (2012a) found altered patterns in CEN and SN activation amongst yoga practitioners, while measuring emotional interference during cognitive tasks. These practitioners showed less reactivity in the right dorsolateral prefrontal cortex (attention control), when viewing negative emotional images. A second finding was that they showed improved functional connectivity between the ventrolateral prefrontal cortex (emotion regulation and impulse control) and SN (amygdala), during a cognitively demanding task in which irrelevant distractor images were presented. A third finding was that while all participants displayed increased SN (amygdala) reactivity when viewing negative emotional pictures, its magnitude predicted decreased positive affect in controls, but not amongst yoga practitioners. In this study it was concluded that yoga practice may promote the ability to selectively implement frontal executive (dependent) strategies to reduce emotional interference during competing cognitive demands. It was also concluded that although practitioners show limbic reactivity to negative emotional stimuli, such reactivity has less downstream effects on later mood states. Accordingly, another controlled study found yoga practitioners to show less CEN (left dorsolateral prefrontal cortex) activation during encoding in a memory task (Gothe et al., 2018).

Studies also indicate yoga to cause structural changes in the brain. One controlled study, for instance, found amongst yoga practitioners increased gray matter in regions related to visual and sensory processing, the somatosensory cortex (mental map of the body), as well as DMN regions related to self-referential processing, memory and cortical responsiveness (Villemure et al., 2015). Another controlled study amongst yoga practitioners found increased gray matter in CEN regions related to higher order cognition as well as other regions related to motor control (Froeliger et al., 2012b).

The experiential learning impact of yoga shows a substantial degree of consistency with the stress buffering account. But while the current studies emphasized long-duration practice, it should be noted that subjects in the Froeliger et al. (2012a) study were doing both yoga and meditation – with the latter practice being an integral part of yoga. Despite this limitation, yoga was found to promote functional connectivity between the CEN and SN (amygdala) during a cognitive distraction task. Here, it was concluded that yoga practitioners do notice negative stimuli, but are less affected by these as amygdala activation becomes more regulated. Other long-term studies seem to support this viewpoint, as yoga practitioners were found to significantly show smaller amygdala volumes (Gotink et al., 2018) as well as a decreased blood flow toward the amygdala (Cohen et al., 2009). In terms of stress buffering, therefore, research indicates yoga practice to support bottom-up processing and alter HPA activation. Furthermore, the findings on improved intra-CEN connectivity as well as specific increases in gray brain mass indicate, albeit indirect, improved self-regulation potential through top-down processing and altered SAM activation. Like aerobic exercises, yogasana practice seems to offer potential for the augmentation of mindfulness, even though the current studies warrant further examination of yoga’s sub-practices and short-term impact. Future studies should therefore aim to parse out independent or interacting effects of each of these sub-practices.

### Both mindfulness and physical exercise impact similar stress regulation pathways

To summarize, the mechanisms described for mindfulness and stress buffering appear to be in parallel with the findings for aerobic exercise and yoga. Both in terms of top-down and bottom-up processing, these practices were found to cause corresponding changes in neural connectivity and contribute to cognitive control over the stress response (see Table 3). Besides being consistent with findings on reduced cortisol, the outcomes on bottom-up regulation may also reflect PSNS function normalization over time. Structural changes in the brain’s architecture due to practice were also found, specifically in regions related to self-regulation. The results for both aerobic exercise and yoga were obtained based on measurements of long-term practice, such as total practice time, practitioner aerobic fitness or activity level – or as a result of repeated single-bout practice. When considering their short-term impact, like Taren et al. (2015) explored for mindfulness, factors such as practice intensity and physical, neurobiological and mental health are likely to moderate single-bout outcomes. Moreover, by corroborating the pathways of stress regulation at an early stage, either aerobic exercise or yoga are likely to contribute to early-stage mindfulness.

Notably, given the neurocognitive overlap with mindfulness, the current outcomes also provide a direction for future study of PE augmented mindfulness. Being consistent with underlying ANS dynamics and stress buffering as described for PE on a physiological level, these findings indicate potential for improved bottom-up regulation. Firstly, the improvements in CEN connectivity indicate changes in top-down processing, while reflecting potential for cognitive control and improvement in sustained – or anchored attention in the short-term. Secondly, the improvements found in SN connectivity point to changes in bottom-up processing; decreased HPA activation can contribute to PSNS activation and stress buffering potential in the short-term. Thirdly, the direct activation of the (parasympathetic) vagus nerve, which may promote other faculties of self-regulation, is likely to contribute to these mechanisms. As mentioned before, the outcomes found in this section were not measured after a single bout of practice, but are rather the result of repeated single-bout practice – and synergistic interaction between its various levels of impact. Further research on PE augmented mindfulness should therefore focus on such interactions and both their short- and long-term potential.

Future research on PE augmented mindfulness could profit from the research methodologies described in this section. These showed extra merit by their inclusion of cognitive, neurocognitive and physiological measures. One avenue to explore the impact of aerobic exercise, yoga or yogasanas is by combining such measurements, when examining their contribution to the mechanisms that underlie mindfulness at an early stage. Future research should therefore aim to compare short-term outcomes on neurobiological, neurocognitive and autonomic functioning; e.g., by incorporating all practices into a controlled research design or by emphasizing methodological comparability.

## Psychophysiological changes due to physical exercise promote neurocognitive functioning

In this section, the direct psychophysiological impact of aerobic exercise and yoga will be examined. In PE augmented mindfulness this impact is expected to play an important role in bottom-up regulation during early-stage mindfulness. On a psychophysiological level, these practices are expected to directly corroborate neurocognition and MA development. The current framework proposes such an impact to be caused by two key-mechanisms. Firstly, these practices cause direct cognitive improvements, as physiological changes promote underlying neurophysiology. Secondly, by causing temporal PSNS reactivation and SNS withdrawal, these practices directly improve self-regulation – as PSNS or vagal tone is increased. These mechanisms will be examined based on cognitive and physiological research literature, with an emphasis on exploring minimal dose–responses that are suitable for PE augmented mindfulness.

The research discussed will emphasize short-term practice outcomes and single-bout dose–responses, in order to chart minimal dose–response characteristics. Where relevant, however, both long- and short-term study outcomes will be described. To ensure optimal neurocognitive benefits, studies in this section adhere to single-bout and repeated practice parameters that cause stress reduction. It is not the scope of this section to deconstruct the underlying physiological processes bottom-up – but rather to give an overview on how practice impact contributes to bottom-up regulation: by leading to direct but temporal improvement in cognition, PSNS or vagal tone and emotion regulation, and associated neurorestorative potential as sleep quality improves.

### Physical exercise practice directly improves cognition

To provide more insight into the temporal cognitive effects of practice, meta-analytic research compared the results of long term (RCT) with short-term (acute effect) studies for aerobic exercise (Smith et al., 2010; Chang et al., 2012) and yoga (Gothe and McAuley, 2015). This approach showed that both practices improve (task positive) cognitive faculties, including attention, processing speed, executive as well as memory functioning – with generally stronger effect sizes for studies that measured acute effects. The research on exercise found for acute effects that practice parameters such as exercise duration, intensity, type of cognitive performance assessed and cardiorespiratory fitness – an indicator of long-term practice – were moderators on these outcomes. Notably, light intensity exercise was found to increase cognition, with its effects subsiding following a delay of more than 1 min. High intensity exercise yielded an opposite pattern. Here, the biggest improvements were found by cognitive tests administered 11–20 min after cessation, with effects subsiding following a longer (>20 min) delay. The researchers concluded that physiological responses to exercise (e.g., cardiorespiratory, metabolic, brain-derived neurotrophic factor, endorphins, serotonin, dopamine) themselves are predictive of its impact on cognitive performance. Nonetheless, the meta-analytic study on yoga did not explore the moderating variables described, although these could be equally relevant for its yogasanas, series of yogasanas or breath exercises. Given the physiological overlap in yoga and aerobic exercise impact (e.g., Ross and Thomas, 2010), similar short-term moderator variables can be expected for yoga.

In addition, studies that focused on single-bout cognitive impact also gauged minimal dose–responses for these practices. For example, 20 min of moderate single-bout aerobic exercise was already found to improve task switching efficiency – which is a core component of executive functioning (Heath and Shukla, 2020). Another study found 10 min of moderate practice to improve oculomotor (eye movement) control – a measure of executive control (Samani and Heath, 2018). For yoga there are indications that 20 min of yoga improves creative problem solving (Bollimbala et al., 2020). Most notably, one controlled study protocol mostly including yogasanas and brief pranayama, found a single 20-min bout of yoga to positively affect working memory and executive functioning (Gothe et al., 2013).

### Physical exercise altered heart rate variability as a marker for improved emotion regulation

Research indicates that improvement in vagal tone due to aerobic exercise or yoga can offer substantial advantages in terms of self- and emotion regulation. These benefits can already be attained following a single bout of practice. Thayer and Lane’s (2000) neurovisceral integration (NVI) model posits that cardiac vagal tone indicates the functional integrity of the neural networks implicated in emotion–cognition interactions. Representing the largest component of the PSNS, the vagus nerve modulates neural interaction between vital organs and the central nervous system (Robertson et al., 2011) and it requires sufficient activation to promote self-regulation over autonomous signal processing (Laborde et al., 2017). Measurement of HRV, according to researchers, is a valid and reliable marker for vagal modulation of the cardiac SA node (Taylor et al., 2010). Variation – based on a cardiogram – is low when there is an inclination toward SNS over-activity, and it becomes higher when parasympathetic health improves. In other words, the healthier the ANS, the faster one is able to switch gears, providing more psychophysiological resilience and flexibility.

Researchers found HRV, not only to be a marker of cardiorespiratory fitness (Dong, 2016), but also predictive to performance in emotion regulation (Williams et al., 2015), and a modulator of feeling states which affect memory retrieval (Fiacconi et al., 2016), executive functioning (Bailey et al., 2015) and decision-making (Dunn et al., 2010; Ramírez et al., 2015). Given that cardiac vagal tone represents the contribution of the parasympathetic nervous system to cardiac regulation, and its positive relation to self-regulation at cognitive, emotional, social, and health levels (Laborde et al., 2017), aerobic exercise or yoga may provide the cardiovascular practice that is required for this vagal activation.

Thus far, meta-analyses show significant HRV changes due to long- and favorable changes due to short-term practice. For aerobic exercise, moderator analysis found indices of HRV to change based on age, sex and previous level of physical activity (Sandercock et al., 2005; Ferreira et al., 2017). For yoga, moderating factors such as practice intensity and frequency were found (Posadzki et al., 2015; Tyagi and Cohen, 2016). One study found yoga practitioners to shift toward parasympathetic predominance after 1 month of 6 days a week hourly sessions that included asana’s, pranayama and meditation (Vinay et al., 2016). By contrast, aerobic practices may require less weekly practice over a longer time period – as it demands more recovery time compared to yoga. Other studies focused on single-bout practice indicate improvement of HRV indices following aerobic exercise (20 min; Terziotti et al., 2001; Hottenrott and Hoos, 2017) or the practice of yoga asana’s (20 min; De et al., 2019). Of note, the latter study emphasized yogasanas rather than a combination of yoga’s practices, indicating that asana practice alone can already impact neurophysiology.

### The neurorestorative impact of physical exercise by improvement of sleep

Research indicates that PSNS activation due to aerobic exercise or yoga can improve practitioner quality of sleep and its neurorestorative potential. Review literature describes these practices to promote sleep quality, but also suggest that their sleep-enhancing effects may not manifest initially (Kline, 2014; Wang et al., 2020). Nonetheless, for a single bout of aerobic exercise, preliminary research found improvements in sleep following around 50 min of moderate or 30 min of vigorous practice (Passos et al., 2010; Wang and Youngstedt, 2014). For yoga, single-bout practice of 30 min reduced stress and improved stress recovery (Benvenutti et al., 2017), while 20 min improved mood (Ensari et al., 2016). Indeed, these findings reflect (temporal) PSNS activation potential, with benefits accordingly; (1) a relaxation response that improves the ability to fall asleep (Benson and Proctor, 2011; Wang and Boros, 2019; Luberto et al., 2020) and (2) PSNS function normalization – of which its restorative components may gradually extend during the deep-sleep stages of a cycle (Chennaoui et al., 2015). By directly improving the underlying physiology of sleep, aerobic exercise or yogasanas can contribute to various neurorestorative processes that support everyday functioning.

Importantly, healthy sleep maintains various neurophysiological processes that support cognition and well-being. As aerobic exercise or yoga can already cause direct PSNS activation and cortisol reduction, single bouts could contribute to a balanced succession of sleep stages within each sleep cycle and, hence, improved neurometabolism. Notably, deep sleep has been associated with specific neurorestorative functions; e.g., neurogenesis in brain regions relevant for emotion regulation and learning (Navarro-Sanchis et al., 2017) and structural changes in the developing brain (Anacker and Hen, 2017).

Research on the neurorestorative impact of sleep particularly confirms the importance of deep-sleep. During the initial, first stage of sleep the brain shows activity linked to muscle memory and learning, whereas the second stage is described as transitory. During the deep-sleep stages three and four, the brain prompts various restorative processes. For example, there is a release of hormones that help regenerate (brain) tissue (growth hormone or GH), guide anti-inflammatory processes (prolactin), enhance immune functioning (cytokines) and stabilize glucose metabolism (mainly cortisol and growth hormone) (Aberg, 2010; Sharma and Kavuru, 2010; Besedovsky et al., 2012; Squire et al., 2012). The ability to enter deep (or slow-wave) sleep is of significant importance for these regenerative and self-healing processes to become operational (Shepalnikov et al., 2012). Initial high levels of cortisol were found to specifically impair these processes, causing an inability to enter deep-sleep and thus sleep deprivation (Hirotsu et al., 2015). Other studies found this type of deprivation related to diminished or even halted releases of GH, which mainly occur during the first sets of sleep cycles (Spiegel et al., 2000, 2005), with high levels of cortisol during sleep related to insulin resistance in the morning, impaired glucose metabolism during the day and debilitated cognitive functioning or attentional lapses (Leproult et al., 1997; Goel et al., 2009; Kim et al., 2015).

Taken together, the practice of aerobic exercise or yoga has been found to directly improve sleep quality. This, in turn, can present short-term benefits for neurophysiology and daily functioning. Although these changes are likely more altered by frequent practice, single-bout practice may already exert a positive influence on the underlying physiological processes described.

### Physical exercise supports neurocognition on a multiple impact level

In support of the PE augmented mindfulness framework, the research on aerobic exercise or yoga points out a relationship between their psychophysiological impact, improved cognitive functioning, parasympathetic activation and improved sleep quality. Firstly, the short-term outcomes provided support for their acute impact on cognition, while for aerobic exercise moderators such as practice duration, intensity, type of cognitive performance assessed, as well as cardiorespiratory fitness were described. Based on the HRV research, similar moderators were found for yogasanas or breath-exercises. Secondly, studies on PSNS activation – as measured by HRV – indicate these practices to directly cause a (temporal) shift in vagal tone and related potential for improved emotion regulation and executive functioning. Thirdly, and this relates to the allostatic changes directly following practice, acute effects of aerobic exercise or yoga may directly contribute to improved sleep and its neurorestorative function. These impact factors can directly define a starting point in early-stage mindfulness and particularly for those who experience ANS dysregulation and related cognitive limitations. These impact factors can also synergistically contribute to well-being, as they are rooted and implied in overall neurocognitive functioning.

Notably, for aerobic exercise or yoga, the practice parameters described in this section correspond to parameters prescribed by the American Heart Association (AHA; also see next section; Wasfy and Baggish, 2016; Piercy and Troiano, 2018). In terms of minimal dose–response characteristics, improvements both in cognition and HRV were found for single-bout practice as short as 20 min for aerobic exercise and 20 min for yogasanas. For the improvement of quality of sleep, minimal dose–responses were studied less extensively. For aerobic exercise, 30 min of vigorous practice was found to improve sleep quality, but future studies may find similar results for shorter and less intense practice. For yoga there are indications that a single bout of 20 min may already contribute to sleep quality. Moreover, sleep and exercise were described to exert substantial positive effects on one another, for which researchers found practice intensity, frequency and cardiorespiratory fitness to be potential moderators (Dolezal et al., 2017). In other words, and this likely applies to yogasanas as well, repeated practice could cause stronger effects on overall quality of sleep. Also, given that practice parameters may vary in accordance to someone’s fitness, or other indicators of long-term practice, such factors can also be considered for PE augmented mindfulness application.

Taken together, the impact factors described indicate aerobic exercise or yogasanas to promote MA development in the short-term. On a practitioner level, these practices offer potential to promote (early-stage) mindfulness meditation; by directly (1) priming the brain for further attention practice, (2) promoting vagal tone and associated improvements in self-regulation, and (3) improvement of sleep and its neurorestorative impact on brain functioning.

## Practice parameters for physical exercise

In PE augmented mindfulness there is an emphasis on PE practice guidelines that can both directly and indirectly promote meditation. To warrant physiological changes that promote neurophysiology and health, there should be adherence to specific practice parameters. Most impact studies, both on long- and short-term aerobic exercise or yoga outcomes, consistently have applied these parameters; e.g., in the neurocognitive or psychophysiological studies described. In the current framework, therefore, these parameters are expected to support the underlying mechanisms proposed and to allow for its clinical potential to be attained.

In particular, single-bout practice parameters are supposed to support underlying neurocognitive processes in mindfulness. A first reason for this is though exercise and yogasanas cause SNS activation, which produce (stress) hormones, SNS activation should be controlled. Practitioners should therefore (a) not exceed the intensity threshold (Hill et al., 2008), which normally lies around 65% of their VO_2_max (total capacity of oxygen take-up), and beyond which cortisol accumulation may start to interfere with cognition (Reyes et al., 2015), (b) let practice intensity increase the heart rate to the point of perspiration in order to prompt physiologic changes that can promote cognition (Boone, 2014), and (c) adhere to minimal practice times, as the intensity threshold can also be exceeded when practice times are too long. A second reason is that these parameters can be matched with the prerequisites for the attainment of flow-state experience. This means that the practice should keep balance between challenge and practitioner skills (both physically and mentally), offer clear goals and unambiguous feedback. In accordance, studies found an inverted U-shaped relationship between flow-state experience and physiological arousal during a task (Peifer et al., 2015) – again supporting the finding that cognition is negatively affected when cortisol levels reach too high.

In addition, also for long-term and consistent aerobic exercises or yogasanas, there are practice parameters that warrant physiological health effects that are supportive to mindfulness. These parameters, also adopted in the protocols of the research described, have been long so propagated by the AHA. Their guidelines state that aerobic exercise should be equated to moderate-intensity aerobic activity for a minimum of 30 min on 5 days, or vigorous-intensity aerobic activity for a minimum of 20 min on 3 days weekly (Wasfy and Baggish, 2016; Piercy and Troiano, 2018). While both aerobic exercise and yogasanas activate glucose metabolism, research also indicates yogasanas to cause sufficiently cardio metabolic change. Because yogasanas can be practiced in such a way to achieve an aerobically intense effort, researchers have examined their cardiovascular and metabolic properties in relationship to AHA guidelines.

In order to match AHA guidelines, research examined dose–responses for yogasanas that could be equated to aerobic practice. For instance, when compared to VO_2_max treadmill baseline testing, it was found that standard compared to fast speed Surya Namaskar (sun salutations) for 8 min could be categorized as low-intensity and moderate- to high-intensity exercise, respectively (Potiaumpai et al., 2016). Likewise, systematic review by Larson-Meyer (2016) describes Surya Namaskar practiced by normal or experienced (perhaps faster paced) practitioners to match moderate or vigorous aerobic intensity, respectively. In addition, while most yogasanas corresponded to light-aerobic activation, specific standing poses (see Larson-Meyer, 2016 for an overview), as well as hatha yoga flow sequences, were found to match the moderate- to vigorous aerobic intensity range. These findings suggest that 30 min of yogasanas, to include multiple standing flow sequences (e.g., Surya Namaskar), specific poses, perhaps alternated with less intense complementary poses, already can create a yogasana practice in the moderate- to vigorous aerobic intensity range.

Taken together, these outcomes offer direction for implementing concrete practice guidelines in PE augmented mindfulness, either by defining minimal dose–responses for exercise, yogasanas or a combination of both.

## Future research on PE augmented mindfulness

Based on this review of literature, mindfulness, aerobic exercise and yoga make claim to similar working mechanisms of self- and stress regulation. This yields four domains of research that should be examined relative to protocolled mindfulness. Firstly, single-bout aerobic exercise or yoga improves the impact of subsequent mindfulness meditation on neurocognitive (e.g., stress buffering, non-reactivity), physiological (e.g., vagal tone), cognitive (e.g., working memory) and psychometric (e.g., MA or sleep quality) outcomes. Secondly, combined repeated practice at minimum corresponding to AHA guidelines, prompts PSNS activation (e.g., vagal tone, allostatic factors, interoceptive ability or sleep) to a faster degree than mindfulness alone. Thirdly, positive effects can be found significantly more to improve for high-stress (e.g., due to ANS dysregulation, related pathology or dysfunctional coping) versus low-stress practitioners. Fourthly, based on the experiential learning effects found for PE, combined practice in the long-term may provide corresponding neurocognitive benefits when compared to mindfulness. Future research in these domains could provide more insight into the working mechanisms that are triggered by PE augmented mindfulness. Such research may also adhere to the methodological considerations described in the previous sections.

One potential underlying mechanism to promote stress regulation in mindfulness lies in what could be termed PE induced vagal tone. This could be best explained along the lines of the mindfulness stress buffering account (Creswell and Lindsay, 2014), which describes vagal toning as a mechanism for SAM axis stress buffering, while vagal tone was also suggested to index CEN (prefrontal) improved functional connectivity (Thayer et al., 2009). This corresponds with the notion that mindfulness promotes the ability for non-reactivity, which has been described to be essential for mindfulness to reach its clinical potential (Whitmarsh, 2013). In other words, improved SAM axis buffering may reflect a mechanism that enables cognitive processes to activate, which subsequently enhances the potential for skilled, thoughtful responses to emotionally arousing situations (Carmody, 2015) – as may be reflected in the experiential learning effects found. Hence, the direct impact of aerobic exercise or yoga on vagal tone (i.e., HRV) and stress physiology may already alter SAM axis activation in the short-term. This impact may therefore be hypothesized to be one of the mechanisms of change in PE augmented mindfulness.

Furthermore, these outcomes on aerobic exercise, yoga or mindfulness can be further examined following the NVI model (Thayer and Lane, 2000; Thayer et al., 2009). Its premises contributed to the stress buffering account and its development; e.g., by describing the relation between inhibitory processes in prefrontal brain regions (CEN), vagal regulation of cardiac stress and health. While proposing a distributed neural control network, allowing for the integration of cognitive, affective, attentional, and autonomic information to guide adaptive goal-directed behavior, a detailed account of this network architecture was not provided. It should be noted, however, as HRV is an indirect measure of cardiac vagal tone, that vagal tone measured on other levels – with different methods – may not always correlate with HRV.

In an update of the NVI model, Smith et al. (2017) proposed an eight level and hierarchical structure of vagal control, discerning different sets of brain and neural mechanisms. This extended NVI model could offer an overarching framework to research the impact of mind-body practices in general and delineate variations in which mindfulness, aerobic exercise, yoga or its sub-practices, and even anaerobic training, affect vagal tone. Notably, each higher level in the hierarchy (see Table 4) is more flexibly recruited to modify vagal tone than the level below, so that high levels (demanding higher cortical activity) will, respectively, be more prominent in vagal output regulation. High levels also integrate a wider range of information (e.g., from exteroceptive perception and memory) in order to regulate current or expected demands on metabolism. The levels described encompass neurocognitive as well as allostatic factors and could help to discriminate physiologic health factors affected by PE – or, for that matter, habitual lifestyle factors. Moreover, here, it is proposed that future studies utilize the PE augmented mindfulness paradigm and its separate sub-practices in order to parse out targeted mechanisms as described by the extended NVI model.

**TABLE 4:** Extended NVI models eight levels and hierarchical structure of vagal control – from high to low level (Smith et al., 2017).

Amplifying, maintaining, or suppressing representations based on current goals
Regulation based on conceptualization of sensory input and past experience
Regulation based on perceptual representation of one’s current somatic/visceral state
Coordinated control of stimulus-driven somatic, visceral and cognitive/attentional responses
Coordinated skeletal-motor, visceral-motor, and endocrine control
Coordinated cross-organ system control
Coordinated cardiovascular control
Intra cardiac control

Each level has different sets of brain and neural mechanisms as well as physiological correlates that contribute to vagal output regulation. Higher levels have more influence on vagal output regulation. The NVI could delineate variations in which mind-body practices (e.g., exercise, yoga, mindfulness or anaerobic training) affect vagal tone.

Furthermore, combinations between aerobic exercise, yoga and meditation can also be expected to promote synergistic effects that promote experiential learning. Though research in this field is limited, one study on the (8 week) MAP protocol found a positive impact for practitioners with major depressive disorder (MDD) (Alderman et al., 2016). This study emphasized the role of hippocampal dysfunction in depression and the specific neurophysiological impact of PE on the hippocampus. MDD practitioners showed significant greater reduction of depressive symptoms and rumination compared to controls following the same program, coupled with altered brain activity in regions related to cognitive control (notably the hippocampal area). Likewise, applications of PE augmented mindfulness (which proposes PE before meditation) may find similar outcomes, as repeated single-sessions may lead to similar synergistic changes over longer time periods. The improvement of vagal tone due to PE, for instance, can also be expected to be a mechanism of change over multiple sessions. The research on the MAP protocol, in line with PE augmented mindfulness, shows that bringing together (existing) neurocognitive and neuropsychological research on different mind-body disciplines can provide a basis for development and research on (pathology specific) treatments.

## Mindful walking as an alternative to aerobic exercise

In the current framework, mindful walking may also be considered as an alternative to aerobic exercise. Both aerobic practices offer advantages, while their differences may be attributed largely to practice volume and intensity parameters. One study examined aerobic exercise and walking programs over 6 months, which were of an equivalent energy cost (Bell et al., 2010), and found both practice types to improve glucose and lipid metabolism – without significant between-group differences. As expected, while both practices improved cardiorespiratory fitness, aerobic exercise was found to be more effective. Furthermore, following single bouts of aerobic exercise of different intensity, research found post-exercise SNS withdrawal to depend on practice intensity, as it was found to dampen the HPA-axis stress response in a dose-dependent manner (Caplin et al., 2021). Likewise, research describes acute and chronic exercise-induced changes in GH levels to be strongly associated with practice intensity (e.g., 30 min on 70% VO_2_max; Gilbert et al., 2008), though sufficient exercise volume may also be required (Sabag et al., 2021). For walking, for instance, meta-analysis found small effect sizes for health factors such as mood, metabolism and cardiovascular parameters (Hanson and Jones, 2015), while researchers associated higher walking pace (e.g., slow, steady/average, brisk) proportionally with higher health outcomes (Stamatakis et al., 2018; Zaccardi et al., 2019).

Notably, within the framework of PE augmented mindfulness, these outcomes indicate physiological impact potential for both practices. Aerobic exercise, given equal single-bout practice times compared to walking, can be expected to prompt improved post-exercise PSNS and vagal activation potential, as well as stress reduction. For walking, decreased cardiovascular activation may result in both less vagal activation potential and release of intensity dependent hormones (e.g., GH) or other physiological components (e.g., neurotransmitters). Following longer duration single-bout walking (e.g., 50 min; Bell et al., 2010), increased physiological changes, vagal activation, and sympathetic withdrawal can be expected. For slow walking the primary mechanisms of change may be prolonged HPA axis activation, general stress hormone reduction and eventual post exercise HPA-axis dampening. For Brisk walking this mechanism may be coupled with an extra parasympathetic vagal response due to increased cardiovascular practice. The degree to which post-walking PSNS activation occurs due to such changes, also in comparison to aerobic exercise, remains to be further investigated.

On a neurocognitive level, less intense exercise such as in mindful walking may save neurophysiological resources for expanded attention practice. While having a different impact on a physiological level, walking and aerobic exercise are similar in that they engage working memory on multiple levels. Following Russell and Arcuri’s (2015) perspective on mindful movement, both practices cause multiple activation of prefrontal brain regions; i.e., increase working memory load, which was found to reduce distractibility (Koshino et al., 2014) and improve sustained attention (Simon et al., 2016). However, prolonged aerobic exercise near the intensity threshold may reduce these benefits or, at least, narrow the intrinsic dynamics of attention practice. Mindful walking, on the other hand, can be physiologically less taxing and may also prompt less working memory load than aerobic exercise, which during practice may promote a wider experiential range similar to meditation. As with the attainment of flow-state awareness, the amount of physiological load (e.g., reflected in stress hormone accumulation), is likely to impact the dynamics of attention function and the impact of increased cognitive load.

Taken together, in the current framework, mindful walking can be concluded to support subsequent mindfulness meditation. However, due to its relatively low practice intensity, a single-bout likely requires longer practice time for post-exercise PSNS activation to occur. Indeed, compared to aerobic exercise, mindful walking was found to cause a more steep decline of cortisol during exercise (Caplin et al., 2021), and potential for vagal toning, while it may lean less on a post-exercise (cardiac) vagal response due to cardiovascular practice. Therefore, mindful walking may provide a better context for breath regulation during practice. Aerobic exercise, on the other hand, may kickstart a stronger post exercise parasympathetic response and other psychophysiological changes, which may offer contrasting therapeutic benefits. When looking at minimal dose responses, 10 min of walking can already improve mood (Edwards and Loprinzi, 2018) and cognition (Suwabe et al., 2018). In PE augmented mindfulness, for these reasons, walking can serve a therapeutic function as an alternative or complementary practice to intense exercise, when sequencing aerobic exercise, yoga and meditation.

## Clinical implications

The PE augmented mindfulness framework proposes practice guidelines (see Table 5), both for the utilization and further study of protocolled or single bout PE augmented mindfulness interventions. Following these guidelines could promote the application of mindfulness in a wide range of mental health treatments, as most forms of pathology involve a substantial degree of ANS dysregulation (e.g., Khalsa et al., 2018). It could benefit both psychological, preventive or complementary treatments as well as multimodal treatment programs; e.g., focused on psychopathology, chronic pain or burn-out. Therapeutic interventions focused on improvement in self-regulation, as utilized in cognitive behavioral therapy (CBT) or acceptance and commitment therapy (ACT), may therefore aim to adopt these practice guidelines. That is to say, under conditions in which the targeted physical exercise and practice of mindfulness are not contraindicated, applications derived from the framework can be advantageous for specific patient groups. PE augmented mindfulness may specifically target pathologies that show a maladaptive stress response, DMN dysfunction and related cognitive disturbances; as suggested by the clinical picture of many disorders (Yamamoto and Hornykiewicz, 2004; Yamamoto et al., 2013), including depression (Thompson et al., 2005; Kung et al., 2010) and anxiety (Gorman et al., 2000) – which are often comorbid to chronic stress disorder (e.g., burnout syndrome; van Dam, 2016).

**TABLE 5:** PE augmented mindfulness: preliminary guidelines for research and protocol development.

Parameters	PE modalities within treatment protocol					
	Light to moderate intense practice		Moderate to intense practice		Minimal practice (estimated) to alter cognition and trigger flow-state experience	
	Aerobic exercise	Yoga	Aerobic exercise	Yoga	Aerobic exercise	Yoga
Intensity	40–60% of VO_2_max^1^	Light intensity^3^	60–80% of VO_2_max^1,2^	More intense^3^	Light to intense	Light to intense
Duration	30 min	30+ min^4^	20 min	20+ min^4^	5–10+ min^5^	5–10+ min^5^
Frequency	5 times weekly	5 times weekly	3 times weekly	3 times weekly	5+ times weekly	5+ times weekly
Density	2 days no practice over total weekly time	2 days no practice over total weekly time	Always a resting day or a minimal practice day after an active day	Always a resting day or a minimal practice day after an active day	5+ days weekly or during non-practice days in the other modalities	5+ days weekly or during non-practice days in the other modalities

^1^Percentages based on the AHA guidelines and the Hill et al. (2008) study.

^2^Practice beyond the ‘intensity threshold’ (± 65% of VO2 Max) causes more SNS strain, but could be an option for specific practitioner groups or part of a build-up schedule.

^3^Intensity in yoga follows from higher intensity poses and holding these poses for a longer time.

^4^Depending on the poses practiced, yoga may require longer practice time than aerobic exercise for similar impact.

^5^Research already indicates potential for cognitive changes after 10 minutes of moderate intensity practice. Depending on the type of practice and degree to which meditation directly follows practice, as well as therapeutic considerations, these times may even be reduced to 5 minutes.

The guidelines pointed out in the current framework can be used to augment existing MBCT- type and MBI protocols or improve single-session interventions. The guidelines described are expected to promote mindful self-regulation (MA) in the short term, which is useful for patients who find sole meditation difficult to practice. In doing so, concurrent characteristics related to emotional wellbeing can be extra facilitated, such as gratitude, self-compassion, self-efficacy, meaning and autonomy (e.g., Ivtzan et al., 2016). Nonetheless, there are many mind-body interventions that increase MA (Xia et al., 2019), which poses the question what type of exercise is best for a specific vulnerable group. Some patients, for instance, can only exercise or move at ‘subthreshold’ intensity, while cardiorespiratory activation already could contribute to their meditations. In these cases (mindful) movement practices may already suffice; e.g., (slow) walking, breathing exercises or light yoga. Particularly interesting is the application of pranayama, notably the alternate nostril breath exercise with retention or the Anuloma viloma technique (Sri Swami Sivananda, 2000), before meditation (Sars, 2022), which can be utilized relatively easily both in individual, group sessions and as a potential alternative to PE itself.

## Conclusion

Taken together, the PE augmented mindfulness framework offers a blueprint for a new approach to mindfulness. Both for clinical and non-clinical practitioner groups. It aims to address both mental and physiological aspects of pathology. The concurrent improvement in (somatic) awareness can promote changes in stress related life-style habits, disturbed circadian health, or generally habits that cause harm to PSNS health (e.g., as defined by the extended NVI model) – which includes patient self-awareness on the detrimental effects of over medicalization in (mental) health care. The framework endorses the notion that mind-body balance precedes actual mental health, analogous to the stages of Maslow’s pyramid that are built upon one another (which raises the question why we even tolerate capitalism driven survival stress). The framework fits into an already ongoing paradigm shift about mental health in general; toward a (multidimensional) system that emphasizes mental, emotional and physical factors, simultaneously, and which values prevention as much as cure. By directly addressing the universal and self-regulatory capabilities of the human mind-body system, the current framework aims to promote a higher accessibility of ‘consciousness-work’ in future health care and beyond.

## Author contributions

DS confirms being the sole contributor of this work and approved it for publication.
